# Adherence to combination antiretroviral therapy among orphaned children in Dar es Salaam, Tanzania

**DOI:** 10.4102/sajhivmed.v20i1.954

**Published:** 2019-08-06

**Authors:** Sabina F. Mugusi, Nassoro Mopei, Omary Minzi

**Affiliations:** 1 Department of Clinical Pharmacology, School of Medicine, Muhimbili University of Health and Allied Sciences, Dar es Salaam, United Republic of Tanzania; 2 Local Government Authority, Dar es Salaam, United Republic of Tanzania

**Keywords:** cART, Adherence, Nevirapine, Tanzania, Orphans

## Abstract

**Background:**

Adherence to combination antiretroviral therapy (cART) among HIV-infected children is often complicated by various factors including medication formulation, dosing frequency, drug toxicities, age and developmental stage, psychosocial and behavioural characteristics of both children and caregivers and can additionally be complicated by being an orphan.

**Objectives:**

This study was aimed at determining the factors and the extent of their influence on cART adherence among HIV-infected orphaned children attending Care and Treatment Centres (CTCs) in Dar es Salaam, Tanzania.

**Methods:**

A cross-sectional study was performed, which assessed adherence in HIV-positive orphaned children aged 2–14 years receiving nevirapine (NVP) based cART for at least 6 months. Data was collected using questionnaires administered to primary caregivers of HIV-infected orphaned children, the review of medical files, and the laboratory measurement of NVP plasma concentrations and CD4 counts. Adherence to cART was determined based on caregivers’ self-report, consistency of clinic attendance and NVP plasma concentrations.

**Results:**

Among the 216 enrolled orphaned children, adherence to cART was found to be 79.6%, 82.9% and 72.2% respectively based on caregivers’ self-report, clinic attendance and NVP plasma levels. Significant reductions in NVP concentrations (< 3 µg/mL) were seen among children with poor immunological outcomes, poor clinic attendance (*p* < 0.05) and were suggested by caregivers’ self-reported adherence (*p* = 0.06). Adherence challenges identified by caregivers included financial constraints (87.5%), lengthy waiting times at clinics (75.5% spent > 2 h at the clinic) and low HIV knowledge among caregivers.

**Conclusion:**

Significant numbers of HIV-infected orphans have poor adherence to cART ranging between 17% and 28% based on different assessment methods. Inadequate caregiver knowledge of HIV/AIDS, long clinic waiting times and forgetfulness were identified as barriers to cART adherence in these orphans.

## Introduction

Three decades on, human immunodeficiency virus (HIV) infection continues to be a global public health problem. The widespread use of combination antiretroviral therapy (cART) has reduced new HIV infections globally, including a 56% drop among children between 2010 and 2016.^1^ Approximately 17 million children have lost one (single orphan) or both parents (double orphan) to this pandemic globally with a staggering 90% of these children living in sub-Saharan Africa.^2^ In Tanzania, there are about 1 million children without parents because of HIV and AIDS; of this, about 40 000 are infected with HIV.^3,4^

Poor adherence to cART among HIV-infected children and adolescents is common and has various contributory causes. Drug-related factors such as formulation of drugs, dosing frequency, toxicities and side effects can affect adherence.^5^ The child’s age and developmental stage, along with psychosocial and behavioural characteristics of children and caregivers, have also been associated with poor adherence to cART.^6,7,8^ In most resource-limited settings, the death of one or two parents has a great effect in terms of financial, material and emotional support in caregiving situations.^9^ In addition to emotional stress, orphans face numerous stressors that could impede their cooperation with treatment and that present significant barriers to maintaining 100% adherence to cART.^10,11^

Adherence to cART suppresses viral replication, delays disease progression and avoids the emergence of viral resistance.^7^ Apart from the regular measurement of the viral load, there is no single adherence measurement tool that has the attributes of being simple to use, non-invasive, sufficiently sensitive, specific and predictive of adherence. Access to viral load monitoring in much of Africa is limited by its cost and general unavailability. Therefore, an alternative multi-method tool may be useful in resource-limited countries. The CD4 cell count has been used as an indirect measure of cART adherence but on its own is nonspecific and too insensitive to provide accurate adherence information.^12,13^ The measurement of blood drug levels, that is, pharmacokinetic or therapeutic drug monitoring, provides a very specific and generally sensitive measure of adherence but is limited due to lack of access in many low and middle-income countries (LMICs). Trough plasma concentration of nevirapine (NVP) of > 3.0 mg/L has been suggested as an indicator of adherence.^14,15^ Mghamba et al. recommended the use of NVP plasma concentration in the assessment of adherence in HIV-infected children as the concentration is a good predictor of adherence.^16^

Factors such as characteristics of a child, caregiver (surviving parent or extended family), cART regimen, social challenges and disclosure of HIV status to the child affect cART adherence among orphans.^17,18,19,20,21,22,23^ Reliable and consistent ways of measuring adherence to therapy would help early detection of patients with poor adherence and allow for immediate and appropriate interventions.^24,25^

## Study objective

This study aims to report the extent of adherence to cART among HIV-infected orphans by assessing caregivers’ self-report, consistency of clinic attendance, CD4 cell counts and NVP plasma levels among HIV-infected orphans and determining factors that may hinder adherence in this population.

## Methods

### Study design, setting and participants

This is a cross-sectional study conducted between June and September 2015 at three urban district specialised paediatric HIV care and treatment centres (CTCs) within the city of Dar es Salaam, Tanzania. These sites had a high paediatric patient enrolment with similar patient population demographics. The study participants were enrolled consecutively as long as they met the inclusion criteria until the required sample was achieved. A study sample of 216 participants was required based on the estimated paediatric cART adherence in Tanzania by Mghamba et al.^16^ The study included HIV-infected orphans (single or double orphaned) aged 2–14 years receiving NVP-based cART for at least 6 months, accompanied by a parent or guardian who gave written informed consent.

### Data collection and study measurements

Nurse counsellors administered a pretested questionnaire to primary caregivers of the HIV-infected orphans which collected information on the sociodemographic and clinical characteristics of children and their primary caregivers. The questionnaire also explored challenges faced by caregivers regarding taking care of the orphans that could potentially lead to poor adherence. A modified indicator-based approach (as described in the World Health Organization manual) was used to measure adherence among HIV-infected orphans.^26^ Using this approach, the two core adherence indicators used to measure adherence in orphans were caregiver self-report and clinic appointment records. Caregiver self-report involved questioning the primary caregiver on the number of doses of antiretroviral therapy that had been omitted in the past 3 days in a non-judgmental manner. Missing one or more doses in 3 days was defined as an equivalent of less than 95% adherence. Retrospective record review of patients’ files was performed to retrieve data on the pattern of clinic attendance of the orphans. Adequate adherence to cART was defined when patients attended the CTC on or before the day of their scheduled appointment or within 3 days of their appointment.^26^

The ages of orphans were grouped into three (2–4, 5–9 and 10–14 years) for descriptive purposes and grouped into two (2–9 and 10–14 years) for the analysis of association. The pattern of CD4 counts over time was also collected using the retrospective record review. Immune status was categorised as good immune status if the percentage of CD4 > 25% for children below 5 years or CD4 count > 500 for children above 5 years.^27^ This pattern of changes in CD4 counts was studied to determine response to cART, as well as using it as a measure of adherence.

A blood sample of 2 millilitres (2 mL) was collected from each child in an ethylene diamine tetra-acetic acid tube. The blood samples were centrifuged immediately to obtain plasma, and these samples were later stored at −80 °C until drug assay. Nevirapine plasma concentration was determined by high-performance liquid chromatography (HPLC) based on the method described by Kappelhoff et al.^28^ This was carried out in the Muhimbili University of Health and Allied Sciences (MUHAS) – Swedish International Development Cooperation Agency (Sida) Bioanalytical Laboratory, Unit of Pharmacology and Therapeutics, School of Pharmacy, MUHAS in Dar es Salaam, Tanzania. Nevirapine concentrations in patient test samples were calculated by a linear regression equation (model) from calibration curve. Nevirapine plasma concentration ≥ 3 µg/mL was categorised as good adherence. This cut-off point was based on the steady-state trough concentration reached in the pharmacokinetic curve for NVP at a dose of 200 mg twice daily.^29^

### Statistical data analysis

Descriptive statistics for categorical sociodemographic characteristics of participants such as orphan sex, orphan status, HIV disclosure, caregiver age, caregiver marital status, caregiver relation to orphan, WHO status, caregiver self-report and clinic attendance were described as frequencies and proportions. For quantitative variables such as orphan age, caregiver age and NVP plasma concentrations, means with standard deviations (s.d.) or medians with interquartile range (IQR) were analysed using independent *t*-test and Chi-square test (χ^2^). The outcome variable was NVP plasma concentration (either below or above 3 µg/mL as a measure of adherence). Univariable and multivariable logistic regression and kappa statistic were used to assess agreement between adherence measures (caregiver report, clinic attendance, CD4 cell count and NVP plasma concentration). The dependent variable was adherence based on NVP plasma concentration. Variables in univariate analysis were included in multivariate analysis to assure that all pertinent and potentially predictive variables were studied. The association between independent and dependent variables was measured using odds ratios (OR) with their corresponding 95% confidence intervals (CI). The significance level for this study was set at a *p*-value of < 0.05. Statistical Package for the Social Sciences (SPSS) version 20 was used for the analysis.

## Ethical consideration

Ethical clearance to conduct the study was obtained from the Institutional Review Board (IRB) of the Muhimbili University of Health and Allied Sciences (MUHAS). Written informed consent was obtained from caregivers of the HIV-infected orphan children aged < 8 years old, and child assent together with caregiver consent was obtained for children aged 8–14 years.

## Results

### Sociodemographic characteristics

A total of 216 HIV-infected orphans were recruited. They were aged between 2 and 14 years (mean ± s.d. age of 9.3 ± 3.3 years) with over 50% of the participants being between the ages of 10 and 14 years. All children were on cART for at least 6 months, with over 94% of the participants being on cART for more than a year. A total of 52 children (24.1%) were double orphans having lost both parents, whereas among the single orphaned children, 37% (*n* = 80) were paternal orphans and 38.9% (*n* = 84) were maternal orphans. The children were under the care of either a surviving parent (38.4%) or a relative (61.6%) with more female primary caregivers than male (80.6% female vs. 19.4% male). Caregivers’ ages ranged between 14 and 70 years (mean age 43.1 [±12.9] years) with a majority being married or cohabiting (51.9%), which reflects a relatively stable family environment. There were two (2) caregivers who were below the age of 18, both being siblings of the orphans. Detailed sociodemographic characteristics are presented in Table 1.

**TABLE 1:** Sociodemographic and clinical characteristics of the patients and caregivers in relation to the nevirapine plasma concentrations.

Variable	NVP plasma concentrations				*p*
	NVP ≥ 3 µg/mL		NVP < 3 µg/mL		
	*n*	%	*n*	%	
**Orphan sex**					
Male	81	75.0	27	25.0	0.362
Female	75	69.4	33	30.6	
**Orphan age**					
2–4	19	82.6	4	17.4	0.398
5–9	62	73.8	22	26.2	
10–14	75	68.8	34	31.2	
**Orphan status**					
Single orphan	121	73.8	43	26.2	0.364
Double orphan	35	67.3	17	32.7	
**HIV disclosure**					
Yes	97	69.3	43	30.7	0.191
No	59	77.6	17	22.4	
**Caregiver sex**					
Male	33	78.6	9	21.4	0.306
Female	123	70.7	51	29.3	
**Caregiver age**					
15–30	16	80	4	20	0.716
31–49	98	71.5	39	28.5	
50>	42	71.2	17	28.8	
**Caregiver marital status**					
Married/cohabiting	83	74.1	29	25.9	0.508
Single	28	65.1	15	34.9	
Divorced/widowed	45	73.8	16	26.2	
**Caregiver education**					
No formal education	9	60	6	40	0.504
Primary education	113	72.4	43	27.6	
Secondary and post-secondary	34	75.6	11	24.4	
**Caregiver relation to Orphan**					
Parent	60	72.3	23	27.7	0.986
Guardian	96	72.2	37	27.8	
**Baseline WHO stage**					
Stage I & II	53	70.7	22	29.3	0.710
Stage III & IV	103	73.0	38	27.0	
**Current Immunological status**					
> 25% or > 500 cells/µL	119	78.3	33	21.7	0.001
< 25% or < 500 cells/µL	31	54.4	26	45.6	
**cART duration**					
< 1 year	10	83.3	2	16.7	0.377
> 1 year	146	71.6	58	28.4	
**Caregiver self-report**					
Good	128	74.4	44	25.6	0.154
Poor	28	63.6	16	36.4	
**Clinic attendance**					
Good	135	75.4	44	24.6	0.021
Poor	21	56.8	16	43.2	

NVP, nevirapine; cART, combination antiretroviral therapy.

### cART adherence measures

Good adherence as assessed by caregiver’s self-report based on not missing a single cART dose in the past 3 days was 79.6%, whereas adherence based on consistent clinic attendance within 3 days of scheduled appointment visit was 82.9%. The analysis of the plasma NVP concentrations showed that 72.2% of the children had good adherence based on the NVP concentrations ≥ 3 µg/mL.

Using NVP plasma concentrations as the more reliable adherence outcome measure compared to the other two qualitative measures, it was found that children with a higher CD4 cell count (> 500 cells/µL or > 25%) had significantly higher plasma concentrations (median [IQR] 6.3 [3.7–9.7] µg/mL) compared to those with lower CD4 cell counts; median (IQR) of 2.8 (0.8 µg/mL – 5.9 µg/mL) (*p* ≤ 0.001). Likewise, patients who had good clinic attendance also had significantly higher median (IQR) plasma NVP concentrations of 5.9 (3.0 µg/mL – 9.3 µg/mL) compared to those with poor clinic attendance (3.6 [1.2 µg/mL – 6.5 µg/mL] *p* = 0.005). Based on caregivers’ self-report, higher NVP plasma concentrations were noted among those who reported good adherence compared to those with poor adherence, 5.7 (2.9 µg/mL – 9.5 µg/mL) and 4.8 (1.6 µg/mL – 6.8 µg/mL) respectively, although these results were not significant (*p* = 0.06). Bivariate analysis showed statistically significant correlations between NVP plasma levels and clinic attendance (*p* = 0.02) and NVP plasma concentrations and immunological status (*p* = 0.001).

The relationship between NVP plasma concentrations and caregivers’ and patients’ demographic/clinical characteristics was measured using univariate logistic regression (Table 2). Disclosure status and orphan status presented notable associations. It was found that patients with disclosed HIV status were less likely to have below the therapeutic plasma NVP concentrations (< 3 µg/mL), compared to undisclosed patients (unadjusted odds ratio, UOR: 0.65, 95% CI: 0.34 to 1.24). Being a double orphan was associated with 1.37 increased risk of having sub-therapeutic plasma NVP concentrations, compared to having at least one surviving parent (UOR: 1.37, 95% CI: 0.69 to 2.68). Confounding bias introduced in the crude association between plasma concentrations and caregivers’ and patients’ demographic/clinical characteristics was also assessed using multivariate logistic regression models. Results from the multivariate models were used to determine the association between NVP plasma concentration, the caregivers’ and patients’ demographic/clinical characteristics after adjusting for each demographic and clinical characteristic. Adjusted associations of disclosure status and NVP concentration was: AOR: 0.64, 95% CI: 0.32 to 1.27 and for orphan status was: AOR: 1.38, 95% CI: 0.62 to 3.05. Table 3 details these univariable and multivariable logistic regression analyses.

**TABLE 2:** Univariable and multivariable logistic regressions looking at the association between NVP plasma concentrations and other adherence measures.

Adherence measure	Univariable regression							Multivariable regression		
	NVP plasma concentration				Crude association			Adjusted association		
	≥ 3 µg/mL *n*	%	< 3 µg/mL *n*	%	Odds ratio	95% CI	*p*	Odds ratio	95% CI	*p*
**Self-reported adherence**										
Good	128	74.4	44	25.6	0.60	0.298, 1.2	0.16	0.62	0.30, 1.27	0.19
Poor	28	63.6	16	36.4	1	-	-	1	-	-
**Clinic attendance**										
Good	135	75.4	44	24.6	0.43	0.21, 0.89	0.02	0.45	0.21, 0.95	0.04
Poor	21	56.8	16	43.2	1	-	-	1	-	-

NVP, nevirapine; CI, confidence interval.

**TABLE 3:** Univariable and multivariable logistic regressions for association between nevirapine plasma concentrations and the caregivers’ and patients’ sociodemographic and clinical characteristics.

Adjusting variable	Univariable regression							Multivariable regression		
	≥ 3 µg/mL *n*	%	< 3 µg/mL *n*	%	UOR	95% CI	*p*	AOR	95%CI	*p*
**Care giver gender**										
Male	33	78.6	9	21.4	1.52	0.68, 3.40	0.31	1.65	0.68, 3.95	0.29
Female	123	70.7	51	29.3	1	-	-	1	-	-
**Caregiver age groups**										
14–49	114	72.6	43	27.4	1.01	0.55, 2.08	0.84	1.11	0.51, 2.47	0.78
50–79	42	71.2	17	28.8	1	-	-	1	-	-
**Caregiver employment status**										
Businessman	74	71.2	30	28.8	1.11	0.61, 2.01	0.74	1.05	0.54, 2.02	0.89
Not businessman	82	73.2	30	26.8	1	-	-	1	-	-
**Caregiver marital status**										
Married	82	73.9	29	26.1	0.82	0.45, 1.49	0.52	0.96	0.496, 1.87	0.91
Not married	74	70.5	31	29.5	1	-	-	1	-	-
**Caregiver education**										
Some education	147	73.1	54	26.9	0.55	0.19, 1.62	0.28	0.58	0.17, 1.95	0.38
No education	9	60.0	6	40.0	1	-	-	1	-	-
**Disclosure status**										
Disclosed	97	69.3	43	30.7	0.65	0.34, 1.24	0.19	0.64	0.32, 1.27	0.21
Not disclosed	59	77.6	17	22.4	1	-	-	1	-	-
**Orphan caregiver relations**										
Parent	60	72.3	23	27.7	0.99	0.54, 1.84	0.99	0.82	0.38, 1.74	0.65
Guardian	96	72.2	37	27.8	1	-	-	1	-	-
**Orphan age group**										
2–9	81	75.7	26	24.3	1.41	0.77, 2.57	0.26	1.40	0.73, 2.68	0.31
10–14	75	68.8	34	31.2	1	-	-	1	-	-
**Orphan status**										
Double orphan	35	67.3	17	32.7	1.37	0.69, 2.68	0.37	1.38	0.62, 3.05	0.43
Not double orphan	121	73.8	43	26.2	1	-	-	1	-	-
**Orphan sex**										
Male	81	75.0	27	25.0	1.32	0.73, 2.39	0.36	1.38	0.74, 2.57	0.32
Female	75	69.4	33	30.6	1	-	-	1	-	-
**WHO clinical stage**										
Stage I and II	53	70.7	22	29.3	0.89	0.48, 1.65	0.71	0.90	0.47, 1.71	0.75
Stage III and IV	103	73.0	38	27.0	1	-	-	1	-	-
**ART duration**										
< 1 year	10	83.3	2	16.7	1.99	0.42, 9.34	0.39	1.21	0.31, 4.73	0.78
> 1 year	146	71.6	58	28.4	1	-	-	1	-	-

ART, antiretroviral therapy; UOR, unadjusted odds ratio; AOR, adjusted odds ratio; CI, confidence interval.

To validate the different adherence assessment methods used of caregivers’ report, clinic attendance and biological outcomes (Immunological outcome) consistency against the actual detected plasma NVP drug concentrations, the sensitivity, specificity, as well as the negative and positive predictive values were calculated. It was concluded that all these methods were highly sensitive (over 80% sensitivity) to predicting levels of adherence among the HIV-infected orphans. Despite the high sensitivity, these methods lacked specificity particularly for the caregivers’ report and clinic attendance. Notable specificity was found with immunological status, which had 50.8% specificity at predicting adherence levels. These methods also proved to have high positive predictive values of over 74% with the immunological status having a positive predictive value of 80.9% (Table 4).

**TABLE 4:** Performance of caregivers’ report and consistency of clinic attendance as compared with NVP plasma concentration for predicting inadequate adherence.

Variable	NVP conc.								
	< 3 µg/mL *n*	%	≥ 3 µg/mL *n*	%	Total *n*	Sensitivity (%)	Specificity (%)	PPV (%)	NPV (%)
**Caregiver report**									
Good	44	25.6	128	74.4	172	82.1	26.7	74.4	36.4
Poor	16	36.4	28	63.6	44	-	-	-	-
**Clinic attendance**									
Good	44	24.6	135	75.4	179	86.5	26.7	75.4	43.2
Poor	16	43.2	21	56.8	37	-	-	-	-
**Immunological status**									
≥ 500 cells/μL	29	19.1	123	80.9	152	82.0	50.8	80.9	52.6
< 500 cells/μL	30	52.6	27	47.4	57	-	-	-	-

NVP, nevirapine; PPV, positive predictive value; NPV, negative predictive value.

### Findings related to the challenges on cART in orphans

The analysis of questionnaires involved the assessment of responses from the caregivers on some of the challenges they face regarding taking care of orphans on cART. Major challenges identified by most caregivers were financial constraints, which include transportation cost to the clinic (87.5% of caregivers incurred transport expenses) and long waiting times at the clinic (75.5% spent more than 2 h at the outpatient HIV clinics). Other challenges faced when bringing the orphan to the clinic include being busy at work (33.8%) and forgetting the appointment dates for drug refills (10.3%). Of those who had missed cART doses, most caregivers attributed the missed doses to forgetfulness (46.7%), being busy at work (15.6%) or being away from home thereby being unable to oversee drug administration (15.6%). Although the knowledge of the caregivers on HIV/AIDS was above average in some aspects, there was lack of knowledge on isolated issues. A total of 91.2% of caregivers did not know that the emergence of drug resistance might result from not taking cART as per instructions. Fewer than half of the respondents could link the contribution of cART to the decreasing incidence of opportunistic infections (44.4%), reduction of viral loads (31%) and the increase in CD4 cell counts (29.2%).

## Discussion

In this study, cART adherence levels were measured using three different measurement tools: caregivers’ self-report, clinic attendance consistency and NVP plasma levels determination. Based on this, adherence to cART was found to be 79.6%, 82.9% and 72.2% by caregivers’ self-report, clinic attendance and NVP plasma levels, respectively. The assessment of adherence based on orphans’ immunological status showed a correlation of 78% between CD4 counts above 500 cells/µL among patients with good adherence to cART. These cART adherence levels among orphans established in this study are still relatively low compared to reported adherence levels in non-orphaned children studies.^23,30,31^ Mghamba et al. in 2012 used the same CTCs to study cART adherence levels in non-orphaned children and obtained higher levels of adherence compared to what was observed in this study.^16^ The difference in cART adherence levels observed between the two study populations could indicate the role of parents in the care of their children and cART adherence.

The assessment of adherence by NVP plasma levels in this study found 27.8% of the orphans to be non-adherent, putting them at a great risk of inadequate virological suppression and subsequent development of drug resistance.^32^ The average non-adherence rate in this study looking at all the different methods of determining adherence is 21.8%, which is similar to other adherence studies showing an average non-adherence rate of around 24.8%.^33^ In a study conducted in North-Western Tanzania, 28.3% of the adult HIV-infected patients were found to have sub-therapeutic plasma antiretroviral drug concentrations. Furthermore, they found that the proportion of patients with sub-therapeutic antiretroviral plasma concentrations had significantly higher viral loads.^34^ Plasma levels have the potential to be highly objective adherence measures and provide unequivocal evidence that medication has been taken. Nevertheless, it should be noted that plasma drug levels are not predictive of adherence behaviour in all patients’ due to factors unrelated to adherence such as drug interactions, individual metabolism variation and poor quality of the drug. Although NVP has a long elimination half-life, the autoinduction of its metabolising enzymes make plasma levels unreliable as a measure of long-term adherence.^35,36^ These factors may affect the plasma concentrations above or below a threshold considered to represent adherence.

Our study found that orphans who had their HIV status disclosed had better adherence compared to those with an undisclosed status. Similar studies from around the region found that children who had their HIV status disclosed were more likely to be adherent to cART.^37,38^ It may be that children who knew their status would be more concerned about their health and therefore understand the rationale behind taking their medications. Our study also found that older children (aged 10–14) were more likely to adhere to cART compared to younger ones (aged 2–9). This finding is in keeping with other studies that showed similar results where older children had better adherence.^38,39^ Older children may have a better understanding of medications and need for adherence, and they can self-medicate. Also, older children have increased independence and improved self-care. Double orphans were also found to have lower adherence compared to single orphan children in this study. This may be related to the caregivers’ motivation, where a non-parental caregiver of a double orphan may be less motivated to care for the orphan compared to a parental caregiver.^40^

The cART adherence levels established in this study by 3-day caregiver recall exceeded the level of adherence as determined by the NVP plasma concentrations. This finding can be in part due to the tendency of patients to provide socially acceptable responses or exaggerated responses to please the provider or because of recall bias.^33,41^ The average sensitivity of good adherence based on self-reported adherence, clinic attendance and immunological parameters was relatively high (83.5%); however, these assessments lacked the specificity. Self-reported adherence is subject to bias with respondents overstating their actual adherence.^42^ Poor clinic attendance may also be due to one or two missed appointments (but not consecutive), which might not correlate with pill counts, where patients may have had extra pills to cover the period.^43^ These results reveal that, whereas high self-reported adherence to cART and clinic attendance rates correlated well with normal therapeutic plasma drug levels among these patients, these tools may still have some limitations while identifying non-adherent patients.

This study also considered challenges caregivers face in taking care of HIV-infected orphan children. Forgetfulness was indicated to be a challenge caregivers face in taking orphan children to the clinic for drug refill and was also implicated as a reason for the child missing at least one dose (46.7%). Several other studies found similar caregiver-related reasons of forgetfulness as a major challenge together with other varied responses as the main reasons for their defaults.^44,45,46^ Distance to treatment centres was also of great concern among the caregivers, with 39.4% of them using over 1 h of travel time to the treatment centre together with high transportation cost. Studies have shown that patients who travelled more than 1 h to hospitals were more likely to be non-adherent.^47,48^ Participants were found to spend unnecessarily long periods of time from arrival at the clinic to getting cART; 75.5% of the study population reported spending more than 2 h at the clinic on account of lengthy queues at outpatient HIV clinics. Long waiting times have also been reported in other studies, and this may discourage patients from going to clinics and remaining in care.^49,50^

Appropriate HIV/AIDS knowledge may play an important role in enhancing cART adherence. The lack of practical knowledge on HIV/AIDS and cART medications, understanding the benefit of cART for children and poor individual adherence to treatments are among the root causes of ineffective cART service delivery. This study revealed a varying lack of knowledge among caregivers on how cART contributes to improvement in health and longevity, where 44.4% of the respondents indicated cART decreased incidence of opportunistic infections. The ramifications of not taking cART were linked to poor immunologic response, increased risk of mortality and rapid progression into AIDS. Potent cART has led to a dramatic decrease in HIV-associated morbidity and mortality.^51^ The majority of caregivers (91.2%) were unaware of the likelihood of drug resistance from inappropriate cART administration. Inappropriate use of cART contributes to the development of HIV drug-resistance.^52^

The variation in adherence by the three different measurement approaches demonstrates the critical importance in the choice of adherence assessment/measure in research and clinical care. The lack of a perfect measure suggests that a composite of multiple measures has a value. Caregiver self-report and patient clinic attendance have a role in this regard. These methods are low-cost, non-invasive and practical and have the potential to provide clinicians with additional insights into the adherence lapses of their patients.

Our study had some limitations. Information collected from the participants was based on self-reports and thus some bias is likely to have been included. Caregiver reported adherence was limited to a 3-day recall bias, and NVP plasma levels only identify poor adherence in the short term. Therefore, this study could not observe change in cART adherence over time nor could actual behaviour be observed. Furthermore, the cross-sectional survey design limits the application of these findings to the individual. The subjects of this study were HIV-infected urban children and orphans, that is, not representative of the general population of Tanzania. During the study period, routine viral load monitoring was not available; hence, a more objective long-term view of adherence could not be assessed.

## Conclusion

This study has demonstrated that significant proportions of HIV-infected orphans on cART have inadequate adherence based on plasma NVP concentrations compared to caregiver self-report. The average adherence rates among the orphans were found to be low (78.2%) based on the three methods of assessment. Differences among the various methods of estimating adherence indicate a need for multiple measurement methods. There is a wide variation in the adherence levels measured by self-reported method, consistency of clinic attendance and NVP plasma determination, indicating that efforts are needed to find out better methods of adherence measurement for both clinical care and research. Inadequate caregiver knowledge on several aspects of HIV/AIDS, unnecessary long waiting time and forgetfulness were identified as barriers to cART adherence among orphans.
